# Advances in industrial microbiome based on microbial consortium for biorefinery

**DOI:** 10.1186/s40643-017-0141-0

**Published:** 2017-02-08

**Authors:** Li-Li Jiang, Jin-Jie Zhou, Chun-Shan Quan, Zhi-Long Xiu

**Affiliations:** 1 School of Life Science and Biotechnology, Dalian University of Technology, Linggong Road 2, Dalian, 116024 Liaoning Province China; 2 Key Laboratory of Biotechnology and Bioresources Utilization, College of Life Science, Dalian Minzu University, Liaohe West Road 18, Jinzhou New District, Dalian, 116600 Liaoning Province China

**Keywords:** Industrial microbiome, Microbial consortia, Biorefinery, Biomass, Bio-based chemicals, Biofuels

## Abstract

One of the important targets of industrial biotechnology is using cheap biomass resources. The traditional strategy is microbial fermentations with single strain. However, cheap biomass normally contains so complex compositions and impurities that it is very difficult for single microorganism to utilize availably. In order to completely utilize the substrates and produce multiple products in one process, industrial microbiome based on microbial consortium draws more and more attention. In this review, we first briefly described some examples of existing industrial bioprocesses involving microbial consortia. Comparison of 1,3-propanediol production by mixed and pure cultures were then introduced, and interaction relationships between cells in microbial consortium were summarized. Finally, the outlook on how to design and apply microbial consortium in the future was also proposed.

## Background

**Fig. 1:**
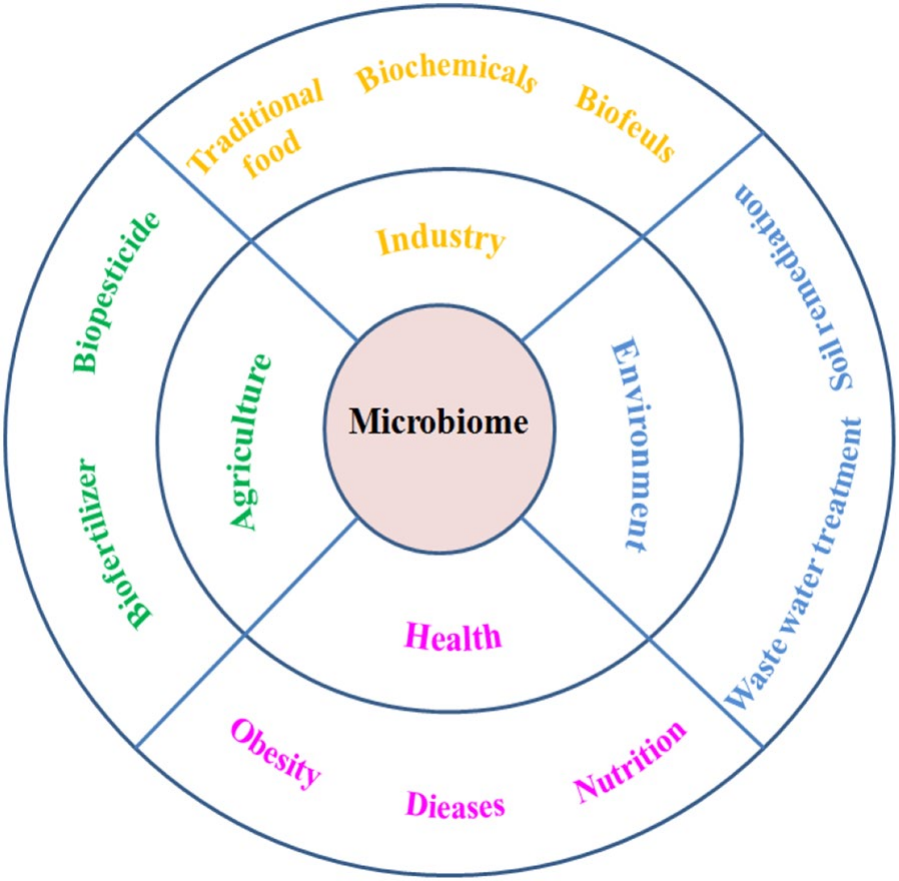
The application of microbiome in industry, agriculture, health, and environment

Human beings have always lived with microbial communities on the earth, but know little about their compositions and functions. Therefore, a group of leading US scientists proposed an Unified Microbiome Initiative (UMI) to research almost all the microbiomes in human, plants, animals, soil, and sea (Alivisatos et al. 2015). They hoped this plan would be paid the same attention with the Precision Medicine Initiative and Brain Initiative in the United States. At the same time, three scientists from Germany, China, and America called for an International Microbiome Initiative (IMI) supported by funding agencies and foundations around the world. They suggested that interdisciplinary experts should cooperate, share standards across borders and disciplines, and realize the integration of resources (Dubilier et al. 2015). Microbiome is a new developing discipline that studies the relationship between microbial consortia in the environment and the growth of animals and plants, as well as human diseases and health. Microbial consortium is referred to microbial community with diverse species on the basis of ecological selection principles. Microbiome can be applied in the fields of industry, agriculture, fishery, medicine, and so on (Fig. 1). The research object of industrial microbiome is microbial consortia applied in food, environment, energy, chemical, and other industrial areas.

The utilization of microbial resources by human has experienced two stages, from naturally mixed culture to pure culture. Human beings have used microbial metabolites for centuries, such as bread, wine, cheese, pickles, and other fermented materials, being provided by fermentation using bacteria and fungi. The bioprocesses were carried out with naturally mixed culture (Sabra and Zeng 2014), which is microbial fermentation by different specified/unspecified microorganisms. In order to avoid contamination of the fermentation process and the product with pathogenic microbes, mixed culture was gradually replaced by pure culture. Without the complicated situation of coexistence of multiple microbes, microbial pure culture allows researchers to be undisturbed for a single strain, and to have a deeper understanding about morphological, physiological, biochemical, and genetic characteristics of microorganisms. Pure culture has built up a milestone for biochemical engineering and modern biotechnology. To date, many bulk biotechnological products such as amino acids, organic acids, antibiotics, and enzymes are almost produced by pure cultures of microorganisms (Sabra et al. 2010). However, about 90–99.8% of the microbes in natural environment cannot be cultured with currently available technologies, and hence cannot be exploited further for biotechnology with pure culture (Streit et al. 2004). The typical problems for biofuels and bio-based chemicals production with pure cultures are the high costs of substrates and product purification, high energy demand for fermentation operation, and high concentrations of by-products in the form of organic acids or alcohols which are toxic to cell growth (Xiu and Zeng 2008; Zeng and Sabra 2011).

In the face of the defects with pure culture, people rethink about the strategies of microbial fermentation. Co-culture is developed based on pure culture, which normally refers to cultures with multiple (mostly two) defined species of microorganisms under aseptic conditions (Sabra et al. 2013). It is a microbial fermentation technology utilizing the different characteristics of microbial growth and metabolism for fermentation (Bader et al. 2010). A typical application of co-culture is the production of 2-keto-l-gulonic acid (2-KLG), the precursor of vitamin C. In the co-culture system, *Ketogulonicigenium vulgare* (small strain) synthesizes 2-KLG from l-sorbose; *Bacillus megaterium* (big strain) as an associated bacterium secretes some metabolites to stimulate the growth of *K. vulgare,* and thus enhances 2-KLG production (Zhang et al. 2010). The researches on fermentation with microbial consortium have been intensive in recent years for overcoming the limitations of pure culture and adapting to the complex substrates and environment. This biotechnology is the industrial application of naturally mixed cultures. On the basis of ecological selection principles, it is able to utilize microbial consortia which can generate a special product spectrum from mixed substrates and reduce the cost of substrates and product purification. Moreover, the processes with microbial consortia have no aseptic requirements (Dietz and Zeng 2014). Microbial consortia usually contain some unknown or non-cultured microorganisms whose effects are unclear. And microbial consortia exhibit strong superiority in the environmental remediation and energy production, such as wastewater treatment with activated sludge and biogas production.

In order to meet the needs of the sustained social and economic development, the industrial biotechnology for a conversion of renewable materials into chemicals and fuels economically has been developed to be an alternative to the traditional chemical industry with high energy consumption and high pollution. Biorefinery has been proposed as one of the key concepts for conversion of renewable materials. Biorefinery is a complex system of sustainable, environment- and resource-friendly technology for material and energy comprehensive use or recovery of renewable raw materials from green and waste biomasses (Kamm et al. 2016). The development of biorefinery is necessary to make various biological products competitive to their equivalent products based on fossil raw materials. The consolidated bioprocessing (CBP) represents an effective and feasible way to implement biorefinery. CBP is referred to integrating all bioconversion reactions in one-step biological process (Minty et al. 2013; Olson et al. 2012). The traditional strategy of CBP is the use of genetically engineered microorganisms focusing on all the required functional genes on one strain. However, many experimental results proved that it was a huge challenge to design and optimize a variety of functions in one strain (Olson et al. 2012). The synthetic biology is also facing the similar challenges in recent years. Compared with CBP based on genetically engineered strains, there are many attractive characteristics of microbial consortia in natural environment, such as composition stability, functional robustness, broad spectrum of substrates, and qualified complex tasks and so on. Therefore, industrial microbiome based on microbial consortium can play an essential role in biorefinery.

## Applications of microbial consortia in industrial fermentations

The application of microbial consortia in traditional foods, such as vinegar, soy sauce, cheese, wine, bread, and pickles, has been recorded for millennia. In the fields of biofuels (biogas, biohydrogen, ethanol, butanol, etc.), bio-based chemicals (1,3-propanediol), biomaterials (polyhydroxyalkanoates), and microbial consortia were also used and studied.

### Biogas

Biogas is a mixed gas containing methane, H_2_, CO_2_, etc., which is converted from organic waste via anaerobic digestion with anaerobic microbial consortia (Bizukojc et al. 2010). Generally, the transformation of organic wastes into biogas is considered to occur in four stages (Sabra et al. 2010). During the hydrolysis phase (Stage I), bio-polymers are degraded into monomers or oligomers which are fermented into volatile organic acids, alcohols, CO_2,_ and H_2_ in the acidogenesis phase (Stage II). In the acetogenesis phase (Stage III), acetic acid as well as some CO_2_ and H_2_ is produced from the molecules formed in Stage II. In the methanogenesis phase (Stage IV), CH_4_ is formed through acetate or CO_2_ and H_2_ by methanogens.

Because of the special growth requirement for some bacteria within microbial consortia, such as a low hydrogen partial pressure, some bacteria are difficult to cultivate using traditional culturing method, such as pure culture. The upflow anaerobic sludge bed (UASB) is the most common type of bioreactors used. In this reactor, methanogenic microbial consortia are present as granules (Diaz et al. 2006). It has been investigated that two-stage process is useful for the treatment of sugar-rich wastewater and bread wastes (Nishio and Nakashimada 2007). In the first stage, bread waste fermented by thermophilic anaerobic sludge at 55 °C was converted to hydrogen and volatile fatty acids (mainly acetate and butyrate), which were then converted to methane in the second stage. Despite of the unsterile process, the thermophilic species from the inoculated microflora were dominating in the hydrogenotrophic stage and the thermophilic process reduced the risk for contamination effectively.

### Hydrogen

As a clean fuel in the future, hydrogen production by fermentation of organic waste has received significant attention in recent years. The main driving force for investigating the production of hydrogen is the economic value of hydrogen, owning to its wide range of applications in the chemical industry, such as synthesis of amines, alcohols, and aldehydes (Li and Fang 2007). And hydrogen is also an ideal fuel, which only produces water after burning. At present, the main difficulty in hydrogen production via microbial anaerobic fermentation is the low yield of hydrogen. The theoretical maximum yield of hydrogen is 4 mol/mol glucose, but in fact the yield of hydrogen from glucose is usually not more than 2 mol/mol due to the consumption of hydrogen by some microorganisms such as methanogens and homoacetogens during the mixed culture (Selembo et al. 2009). It has been proved the pre-treatment by alkali, acid, or heat to make the above hydrogen-consuming microorganisms inactive, and the effect of heat treatment to be best except to homoacetogens (Oh et al. 2003). Due to the complexity of microbial consortium, the intracellular metabolic pathway of hydrogen is also more complex. Lee et al. (2009) developed the first model for predicting community structure in mixed-culture fermentative biohydrogen production through electron flows and NADH_2_ balances. The clone-library analyses confirmed the model prediction, and hydrogen was produced at pH 3.5 only via the pyruvate decarboxylation-ferredoxin-hydrogenase pathway in microbial consortium. This model could easily assess the main mechanism for hydrogen formation and the dominant hydrogen-producing bacteria in mixed culture. Rahul et al. (2012) evaluated the potential of bioconversion of crude glycerol to hydrogen by an enriched microbial community from activated sludge. Hydrogen yield from raw glycerol was almost 1.1 mol-H_2_/mol glycerol consumed under optimal conditions (pH 6.5, 40 °C and 1 g/l raw glycerol).

### Ethanol

Ethanol is an important alternative of gasoline fuel, with the advantages of cheap, clean, environment-friendly, safe, and renewable fuel. At present, the research focused on the conversion of non-food materials, such as lignocellulose to ethanol. The main constituents of lignocellulosic hydrolysates are hexoses (glucose, mannose, galactose, etc.), pentoses (xylose, arabinose, etc.), and several toxic by-products such as phenol, acid, and aldehyde (Eiteman et al. 2008). The traditional pure culture by *Saccharomyces cerevisiae* could not convert mixture of hexoses and pentoses effectively. Du et al. (2015) selected a consortium (named HP) from 16 different natural bacterial consortia, and HP consortium exhibited relatively high ethanol production (2.06 g/l ethanol titer from 7 g/l α-cellulose at 55 °C in 6 days). They found that the community composition affected the performance of producing ethanol from cellulose. Recent studies have proved that natural microbial consortia can produce a variety of cellulases, in order to adapt the degradation requirements of different lignocelluloses. Three new anaerobic gut fungi (*Anaeromyces robustus*, *Neocallimastix californiae,* and *Piromyces finnis*) isolated from herbivores produced the biomass-degrading enzymes which exhibited strong ability to degrade lignocellulose. The relative activity for hydrolysis of xylan with these enzymes especially secreted by *Piromyces finnis* was threefold more than those optimized commercial preparation from *Aspergillus* (Solomon et al. 2016). Thus, cellulosic ethanol production by microbial consortia is a promising method.

### Butanol

Butanol, a four-carbon primary alcohol, is not only an important bulk chemical feedstock, but also a promising next-generation liquid fuel because of its superior characteristics over ethanol, such as higher energy content, less hygroscopicity, better blending ability, and an energy density closer to that of gasoline (Dürre 2007). However, to date, bio-production of butanol is still not economically competitive with petrochemical production because of its major drawbacks, such as high cost of the feedstocks, low butanol concentration in the fermentation broth, and low-value by-products, i.e., acetone and ethanol (Gu et al. 2011). In order to reduce the cost of the feedstocks, biosynthesis of butanol from lignocelluloses gained popularity in recent years. Microbial conversion of lignocellulosic biomass requires multiple biological functionalities, including production of saccharifying enzymes (cellulases and hemicellulases), enzymatic hydrolysis of lignocellulose to soluble saccharides, and metabolism of soluble saccharides to desired products (Zuroff and Curtis 2012).

Consolidated bioprocessing has been suggested as an efficient and economical method of producing butanol from lignocellulose through simultaneous hydrolysis and fermentation with cellulolytic microorganisms and solventogenic bacteria in one bioreactor (Olson et al. 2012). In the consortium, microorganisms may develop the potential for synergistic utilization of the metabolic pathways from interspecies. It was very difficult to produce butanol efficiently from lignocellulose directly by pure culture. Wen et al. (2014) constructed a stable artificial symbiotic consortium by co-culturing a cellulolytic, anaerobic, butyrate-producing mesophile (*Clostridium cellulovorans* 743B) and a non-cellulolytic, solventogenic bacterium (*Clostridium beijerinckii* NCIMB 8052) to produce solvents by consolidated bioprocessing with alkali extracted deshelled corn cobs (AECC) as the sole carbon source. Under optimized conditions, the co-culture degraded 68.6 g/l AECC and produced 11.8 g/l solvents (2.64 g/l acetone, 8.30 g/l butanol, and 0.87 g/l ethanol) in less than 80 h.

### Polyhydroxyalkanoates

Polyhydroxyalkanoates (PHAs) are polyesters, a kind of natural macromolecule biomaterial, which are synthesized and stored within the cell by various microorganisms. PHAs have been recognized as good candidates for biodegradable plastics because of their similar properties to conventional plastics and their complete biodegradability (Lemos et al. 2006). Industrial production processes are based on the use of pure cultures of microorganisms in their wild form or recombinant strains (Vandamme and Coenye 2004). However, due to the pure substrates utilized and the sterile operation of the production process, the cost of PHA production with pure culture is still too high to become a competitive commodity plastic material. Therefore, in order to reduce the cost of PHA production, the interest in the use of mixed cultures for PHA production has increased in recent years (Dias et al. 2006). The production of PHA by mixed cultures could use renewable carbon sources based on agricultural or industrial wastes, and operate under non-sterile condition, which reduce the cost of substrate and equipment investment significantly. Moita et al. (2014) investigated the feasibility of PHA production by a mixed microbial community using crude glycerol as feedstock. The results showed that crude glycerol could be used to produce PHA without any pre-treatment step, leading to the overall production process more economically competitive, reducing polymer final cost.

## Comparison between pure culture of single strain and mixed culture of microbial consortia

Industrial 1,3-propanediol (1,3-PD) production has attracted attention as an important monomer to synthesize a new type of polyester, polytrimethylene terephthalate (PTT), and the market demand is increasing year by year (Zeng and Sabra 2011). The traditional microbial fermentation to produce 1,3-PD is pure culture. This biotechnological method includes wild-type bacteria conversion of glycerol to 1,3-PD and gene-modified bacteria conversion of glucose to 1,3-PD directly (Chatzifragkou et al. 2011; Jolly et al. 2014; Metsoviti et al. 2013; Nakamura and Whited 2003). A surplus of crude glycerol has occurred due to large production of biodiesel; therefore, the conversion of crude glycerol into 1,3-PD was paid more and more attention. Crude glycerol usually contains many impurities such as alcohol, salts, esters or lipids, and pigments, so that it needs to be purified before used for pure culture, no doubt increased the cost of production (Johnson and Taconi 2007).

Up to date, most researches have focused on strain screening (Metsoviti et al. 2012a, b; Raghunandan et al. 2014; Rodriguez et al. 2015), genetically engineered strains (Nakamura and Whited 2003), fermentation optimization of 1,3-PD (Jun et al. 2010; Sun et al. 2010), etc., which were all based on pure cultures. The fermentation based on pure culture usually requires strict aseptic operation and purified substrates, resulting in the high cost of biological production of 1,3-PD. At the same time, in order to balance the intracellular redox state and to supply ATP during microbial production of 1,3-PD, various by-products were produced, such as acetic acid, lactic acid, succinic acid, and other organic acids as well as alcohols. The accumulation of these by-products often inhibits the growth of cells, competes for NADH against the 1,3-PD pathway to reduce the yield of 1,3-PD from glycerol, and brings difficulties for the separation and purification of target product (Xiu and Zeng 2008).

Compared with pure culture, specific advantages of fermentation with microbial consortia include the following: ① the possibility of utilizing cheaper or mixed substrates (e.g., whey, molasses, lignocellulose, and raw glycerol); ② the synergies of different enzymatic systems and combination of metabolic pathways of different microorganisms that can result in more efficient utilization of substrates and a narrow production spectrum contributing to product purification and reducing the cost; ③ due to the high microbial diversity, the operation with microbial consortia has no sterile requirement which will lower the production cost (Sabra and Zeng 2014). Thus, biotechnology based on microbial consortia could become an attractive addition or alternative to traditional biotechnology based on pure culture for the production of chemicals in industrial biotechnology (Sabra et al. 2010).

In order to overcome the shortcoming of pure culture, and reduce the cost of biological production of 1,3-PD furthermore, the fermentation with microbial consortia has been intensively studied in recent years (Dietz and Zeng 2014; Gallardo et al. 2014; Kanjilal et al. 2015; Liu et al. 2013; Temudo et al. 2008). The biological production of 1,3-PD based on pure culture of single strain was compared with that based on mixed culture of microbial consortia (Table 1). Dietz and Zeng (2014) selected microbial consortia from sludge of wastewater treatment plant. 1,3-PD can be produced as the main product in this mixed culture with typical organic acids such as acetic and butyric acids as by-products. The yield was in the range of 0.56–0.76 mol 1,3-PD/mol glycerol consumed depending on the glycerol concentration. A final product concentration as high as 70 g/l was obtained in fed-batch cultivation with a productivity of 2.6 g/l h. This study showed that 1,3-PD production in mixed culture achieved the same levels of product titer, yield, and productivity as in typical pure cultures, especially without sterile requirement. Szymanowska-Powalowska et al. (2013) isolated bacterial strains with capability of the utilization of by-products such as butyric acid and lactic acid. The co-culture of *Clostridium butyricum* DSP1 producing 1,3-PD and *Alcaligenes faecalis* JP1 utilizing organic acids increased the volumetric productivity (1.07 g/l h) and yield of 1,3-PD (0.53 g/g). Moreover, the only by-product present was butyric acid at a concentration below 1 g/l, which significantly reduced the cost of extraction and purification for the target product. This new type of mixed culture provides a new solution to separate and purify target products in the process of bio-based chemicals production.

**Table 1:** Comparison of 1,3-propanediol production by microbial consortia and single strain

Inoculum	Fermentation type	Glycerol type	1,3-PD (g/l)	Yield (mol/mol)	References
Pure culture of single strain					
*Klebsiella pneumoniae* DSM 4799	Fed-batch	Raw	80.20	0.54	Jun et al. (2010)
*Klebsiella oxytoca* M5al	Fed-batch	Pure	83.56	0.62	Yang et al. (2007)
*Citrobacter freundii* FMCC-B 294	Fed-batch	Raw	68.10	0.48	Metsoviti et al. (2013)
*Clostridium butyricum* AKR102a	Fed-batch	Raw	93.70	0.63	Wilkens et al. (2012)
*Lactobacillus reuteri* ATCC 55730	Fed-batch	Pure	65.30	0.81	Jolly et al. (2014)
Mixed culture of microbial consortia					
Organic soil	Batch	Raw	3.76	0.65	Liu et al. (2013)
Wheat soil	Batch	Pure	1.71	0.69	Selembo et al. (2009)
Sludge	Batch	Raw	15.21	0.51–0.76	Dietz and Zeng (2014)
	Fed-batch	Raw	70.00	0.52–0.56	
Granular sludge	Continuous	Pure	10.74	0.52	Gallardo et al. (2014)
Marine sludge	Batch	Pure	81.40	0.63	Xiu et al. (2015)
	Fed-batch	Pure	72.15	0.70	

In the past few years, our lab selected facultative anaerobic microbial consortia from sludge in Dalian seashore. 16S rRNA gene amplicon high-throughput sequencing was performed to investigate the bacterial composition of microbial consortium DL38, and it was found that the most abundant organisms belonged to *Enterobacteriaceae* (95.57%), followed by *Enterococcaceae* (2.10%), *Moraxellaceae* (1.21%), and *Streptococcaceae* (0.64%). The results showed that mixed culture with microbial consortium DL38 (Genbank accession number: SRP066989) possessed excellent substrate tolerance and narrow product spectrum, leading to the biological production of 1,3-PD more attractive and competitive. The yield was in the range of 0.57–0.70 mol 1,3-PD/mol glycerol consumed, which depended on the glycerol concentration. The initial glycerol concentration of batch fermentations with microbial consortium DL38 was up to 200 and 81.40 g/l of 1,3-PD was obtained with yield 0.63 mol/mol. In batch fermentation, a small amount of by-products were produced, especially no 2,3-butanediol was detected in favor of 1,3-PD purification (Jiang et al. 2016).

Compared with pure culture of single strain, mixed culture of microbial consortium normally showed higher efficiency or productivity and substrate tolerance. This is undoubtedly attributed to the interactions among cells in microbial consortium as discussed in the next section, although they are seldom known clearly. On the other hand, the metabolites or intermetabolites (even amino acids and nucleotides), or coenzymes (e.g., NADH/NADPH) or cofactors (e.g., ATP) produced from one strain might regulate the growth and metabolism of another strain. Besides the mechanism of mixed culture, the stability of microbial consortium structure during fermentation is also an important problem in industrial process. Some researchers aimed to bring ecological and evolutionary concepts to discussion on this question (Escalante et al. 2015). They pointed out that the system composed of cooperative consortia may be collapsed by cheaters arising during evolution (Diggle et al. 2007). We need to determine the primary strains in microbial consortia by incorporating evolutionary and ecological principles, and to design evolutionarily stable and sustainable systems by artificial structure of microbial consortia on the basis of biotechnological demand.

## The interactions among cells in microbial consortia

In microbial consortium, there exist not only intraspecies interactions among the same species of microbial cells, which usually accomplish through quorum sensing (QS), but also interspecies interactions between different species cells, such as mutualism, competition for nutrition in the same ecological environment. These mutual effects based on metabolites will affect metabolisms and the yield of target product in the fermentation process.

### Quorum sensing

Quorum sensing (QS) is characterized by communication information relying on bacterial density, leading to the realization of coordinated behaviors through responsive gene expression. The microbial cells can release some specific signal molecules and detect the change of their concentrations spontaneously, thus coordinating behaviors upon the establishment of a sufficient quorum (Schertzer et al. 2009). *N*-acyl-homoserine lactones (AHLs) are often used by Gram-negative bacteria as the QS signals (Williams 2007). In stark contrast to Gram-negative bacteria, Gram-positive bacteria make and transport autoinducing peptides (AIPs) as communication signals (Parsek and Greenberg 2000). Each species of Gram-negative or Gram-positive bacteria produces a unique AHL (or a unique combination of AHLs) or AIPs. As a result, only the members of the same species recognize and respond to it (Federle and Bassler 2003).

The species-specific QS described above promotes intraspecies communication and apparently allows self-recognition in a mixed population. In such situations, bacteria also develop mechanisms to detect the presence of other species, and the signals of AI-2 (autoinducer-2) family are used for interspecies communication (Pereira et al. 2013). The evidence for the existence of AI-2 came from studies of the Gram-negative bioluminescent shrimp pathogen *Vibrio harveyi* (Bassler et al. 1997). AI-2 is synthesized by an enzyme called LuxS. However, the gene *luxS* is present in the genomes of a wide variety of Gram-negative and Gram-positive bacteria. Therefore, every bacterium containing a functional *luxS* gene is capable of producing an activity detected by an AI-2-specific *V. harveyi* reporter strain (Federle and Bassler 2003). AI-2 is a more universal signal that could promote interspecies bacterial communication. Quorum sensing is a key process in natural microbial interactions (Miller and Bassler 2001), and plays an important role in controlling virulence factor production, biofilm formation, improving microbial stress resistance, etc. (Park et al. 2014; Lin et al. 2016; Gambino and Cappitelli 2016). A biofilm is an group of microorganisms in which cells stick to each other and/or adhere to a surface. These adherent cells are frequently embedded within a self-produced matrix of extracellular polymeric substance (EPS). Biofilm formation can significantly improve microbial tolerance for oxygen or substrate or toxic/inhibitory substances. For example, the dissolved oxygen is consumed by one community member in biofilm, and an oxygen gradient can be established to create suitable microenvironments for anaerobic microbes (Gambino and Cappitelli 2016).

### Mutualism and synergism

Mutualism refers to benefit of two or more species to one another when living together, but both of their lives will be affected badly and even die when separated. There are numerous examples of mutualisms in the fermentation processes with microbial consortia. For instance, the relationship between archaea and bacteria is mutualism during the process of anaerobic fermentation to produce methane. Stolyar et al. (2007) first used stoichiometric models through flux balance analysis to analyze mutualistic metabolite exchange between a sulfate reducer *Desulfovibrio vulgaris* and methanogen *Methanococcus maripaludis*. This study can accurately predict the relative abundances of *D. vulgaris* and *M. maripaludis* in an experimental co-culture. Shou et al. (2007) constructed a synthetic obligatory cooperative system, termed CoSMO (cooperation that is synthetic and mutually obligatory), which consists of a pair of auxotrophic yeast strains, each supplying an essential metabolite to the other strain. However, this reciprocal interaction can readily collapse, due to the evolution of “cheater” individuals that receive the benefit of the facilitation without contribution (Nowak 2006). This potential meltdown caused by cheater can be overcome or delayed depending on environmental spatial structure. The physical structure of the environment can limit the spread of cheating genotypes (Hammerschmidt et al. 2014). Synergy is one form of microbial mutualism, in which metabolites produced by one species or genotype affect the growth of other species (Escalante et al. 2015). Synergy interactions are commonly demonstrated in numerous biotechnology studies including consolidated bioprocessing of cellulose coupled with biofuel production (Du et al. 2015) and an organic acid-consuming community member scavenges inhibitory by-products from a producer population (Bizukojc et al. 2010). Kato et al. (2004) isolated two strains from the compost: one was *Clostridium straminisolvens* CSK1 which was able to degrade cellulose efficiently under anaerobic conditions; the other one was an aerobic non-cellulolytic bacterium. They successfully constructed a bacterial community with effective cellulose degradation by mixing the above two strains. The mixed culture indicated that the non-cellulolytic bacteria essentially contribute to cellulose degradation by creating an anaerobic environment, consuming metabolites, and neutralizing pH.

### Competition and antagonism

Competition for limited natural resources within a microbial community is known as the selective force that promotes biosynthesis of antimicrobial compounds. Recently, it was shown that these antimicrobial molecules produced in nature are not primarily used as weapons for competition but as tools of communication that may regulate the homeostasis of microbial communities (Hibbing et al. 2010; Yim et al. 2006, 2007). For example, lactacin B produced by *Lactobacillus acidophilus* would be increased when this strain was co-cultured with the yogurt starter species *Streptococcus thermophilus* and *Lactobacillus delbrueckii* subsp. *Bulgaricus* (Tabasco et al. 2009). Antagonism is an interspecies interaction in which one species adversely affects the other one without being affected itself. It frequently occurs in food fermentations and inhibits the growth of spoilage organisms (Bas et al. 2006).

The interaction among cells in microbial consortium plays an important role to the stability of bacterial community. Recently, some researchers used mathematical models to prove that synergy between different types of microbial cells would disrupt the ecosystem stability of microbial consortium. Moreover, the competitive relationship between probiotics would offset the instability caused by the microbial diversity through negative feedback, and keep the intestinal ecosystem stable (Coyte et al. 2015). Many evidences from ecological perspectives also showed that the evolution of cheaters made the mutualism interaction more fragile than competition (Nowak 2006; Hammerschmidt et al. 2014; Escalante et al. 2015). Thus, the competitive relationship seems to be more conducive for maintaining the stability of microbial consortium.

## Perspectives

Natural microbial consortia hold many appealing properties in one bioprocess, such as stability, functional robustness, and the ability to perform complex tasks (Sabra and Zeng 2014). Inspired by the powerful features of natural consortia, there are rapidly growing interests in engineered synthetic consortia for biotechnology applications (Zuroff and Curtis 2012; Bernstein and Carlson 2012). Brenner et al. (2008) reviewed researches on engineered microbial consortia by designing the communication between different microorganisms. These engineered microbial consortia can be used to study the interspecific interaction relationship (such as symbiosis, competition, and parasitism) in the smallest consortium. In addition, mathematical models can also be used to describe the defined microbial consortium, and used for development and validation of the more complex systems (Bizukojc et al. 2010). In the application of industrial biotechnology, it is more attractive and more promising to screen desired microbial strains from nature and put them together to execute new function. As people actively explore and understand the relationship of the microecology, microbial consortia will be developed and applied in many fields such as industry, agriculture, and food. In order to design and develop a successful process, it is necessary to understand the precise role and the overall contribution of each microorganism to the fermentation process. This knowledge is crucial to an inoculum with a defined co-culture or a mixture of undefined microbial consortium. There are many challenges needed to be faced in fermentation with microbial consortium, such as population dynamics and flux analysis of different species in the same reactor, the interrelationships between species, and the consistency and stability of inocula of microbial consortium during bioreactor scale-up. The most promising method for the determination of population dynamics is the molecular biological one based on the analysis and differentiation of microbial DNA, such as sequencing and metagenomics (Röske et al. 2014). A great deal of information can be gleaned from even very complex microbial communities (Spiegelman et al. 2005). Metabolic networks and stoichiometric models can serve not only to predict metabolic fluxes and growth phenotypes of single organism, but also to capture growth parameters and composition of simple bacterial community (Stolyar et al. 2007; Sabra et al. 2015). The small microbial consortium with several and definite strains has good application prospect, which can be used as a model system in the development of methods and techniques, and is beneficial to use synthetic biology to design microbial consortia. These defined co-culture system would facilitate our understanding of the simultaneous involvement of several different microbial interactions in one and the same industrial process and controlling them (Goers et al. 2014). At the same time, the consistency and stability of inocula of microbial consortium would be maintained if the microbial behavior is understood. Therefore, the thorough research about industrial microbiome based on microbial consortium has not only profound theoretical significance, but also more extensive application potential, and can be of more benefit for humanity.

## Abbreviations

1,3-PD: 1,3-propanediol
2,3-BD: 2,3-butanediol
PHAs: polyhydroxyalkanoates
ATP: adenosine triphosphate
*K. pneumoniae*: *Klebsiella pneumoniae*
*K. vulgare*: *Ketogulonicigenium vulgare*
*V. harveyi*: *Vibrio harveyi*
*D.vulgaris*: *Desulfovibrio vulgaris*
*M. maripaludis*: *Methanococcus maripaludis*
CBP: consolidated bioprocessing
AECC: alkali extracted deshelled corn cobs
QS: quorum sensing
AHLs: *N*-acyl-homoserine lactones
AIPs: autoinducing peptides
AI-2: autoinducer-2 family
